# Limb-Sparing Reconstruction for Pediatric Osteosarcoma: A Case Report of Shoulder Preservation With Proximal Humerus Anatomical Mega Prosthesis and Review of the Literature

**DOI:** 10.7759/cureus.84261

**Published:** 2025-05-16

**Authors:** Muhammed Yusuf Afacan, Arın Celayir, Cansu Elibollar, Mahmut K Ozsahin, Huseyin Botanlioglu

**Affiliations:** 1 Department of Orthopaedics and Traumatology, Istanbul University-Cerrahpasa, Cerrahpasa Faculty of Medicine, Istanbul, TUR

**Keywords:** chemotherapy, limb-salvage surgery, mega prosthesis, osteosarcoma, proximal humerus

## Abstract

Osteosarcoma of the proximal humerus presents significant challenges due to the proximity of critical neurovascular structures. This case report describes the management of an eight-year-old male diagnosed with high-grade intramedullary osteosarcoma of the proximal humerus with axillary lymph node involvement. Following neoadjuvant chemotherapy, the patient underwent wide resection, axillary lymph node dissection, and reconstruction using a proximal humerus anatomical mega prosthesis, followed by adjuvant chemotherapy. Radiotherapy was not required. Early postoperative range of motion was considerably good, with satisfactory shoulder and elbow function achieved. At the 1.5-year follow-up, there was no evidence of local recurrence or metastasis, and the prosthesis remained stable. This report demonstrates that limb-sparing surgery with prosthetic reconstruction offers favorable oncological and functional outcomes and may represent a preferable alternative to vascularized fibula grafting in pediatric patients with proximal humeral osteosarcoma.

## Introduction

Osteosarcoma is the most common primary malignant bone tumor in children and adolescents, accounting for approximately 20% of all primary bone malignancies. It primarily arises in the metaphyseal regions of long bones, with the distal femur and proximal tibia being the most frequently affected sites [1]. However, the proximal humerus represents the third most common location, constituting about 10-15% of cases [2]. The involvement of the proximal humerus poses unique diagnostic and therapeutic challenges due to its anatomical complexity and functional significance in upper limb mobility [2]. Patients with osteosarcoma of the proximal humerus typically present with progressive pain, localized swelling, and, in some cases, reduced range of motion [3]. Unlike osteosarcomas of the lower extremities, those occurring in the proximal humerus are less likely to cause pathological fractures but may significantly impact shoulder function. Early diagnosis relies on a combination of clinical assessment, radiographic imaging, and histopathological confirmation. MRI is essential for assessing tumor extent and neurovascular involvement, while PET-CT is useful for detecting distant metastases, seen in about 20% of cases at diagnosis [4]. 

The poor prognostic factors for osteosarcoma include the presence of metastasis, including skip metastasis, high tumor grade, and poor chemotherapy response with less than 90% necrosis. Tumors located in the pelvis or spine are generally associated with better prognoses, whereas tumors greater than 10 cm in size and the presence of a pathological fracture are negative prognostic indicators. Additionally, male gender and elevated levels of alkaline phosphatase (ALP) and lactate dehydrogenase (LDH) are also associated with poorer outcomes.

The management of proximal humeral osteosarcoma typically follows a multimodal approach, including neoadjuvant chemotherapy, surgical resection, and adjuvant chemotherapy [5]. Limb-salvage surgery is often preferred over amputation, with options such as wide resection followed by reconstruction using allografts, endoprostheses, or biologic techniques [6]. However, achieving negative surgical margins while preserving shoulder function remains a significant challenge, particularly in cases with neurovascular involvement or extensive soft tissue invasion. Additionally, axillary lymph node metastasis, though rare, has been reported and may indicate a more aggressive disease course [6].

This article discusses a case of high-grade intramedullary osteosarcoma in the proximal humerus, highlighting the diagnostic challenges, surgical strategies, and long-term outcomes following tumor resection and reconstruction with a proximal humerus anatomical mega prosthesis. The primary aim of this report is to emphasize the feasibility and effectiveness of limb-salvage surgery using a tumor prosthesis for achieving both oncological control and functional preservation in pediatric osteosarcoma.

## Case presentation

**Figure 1:**
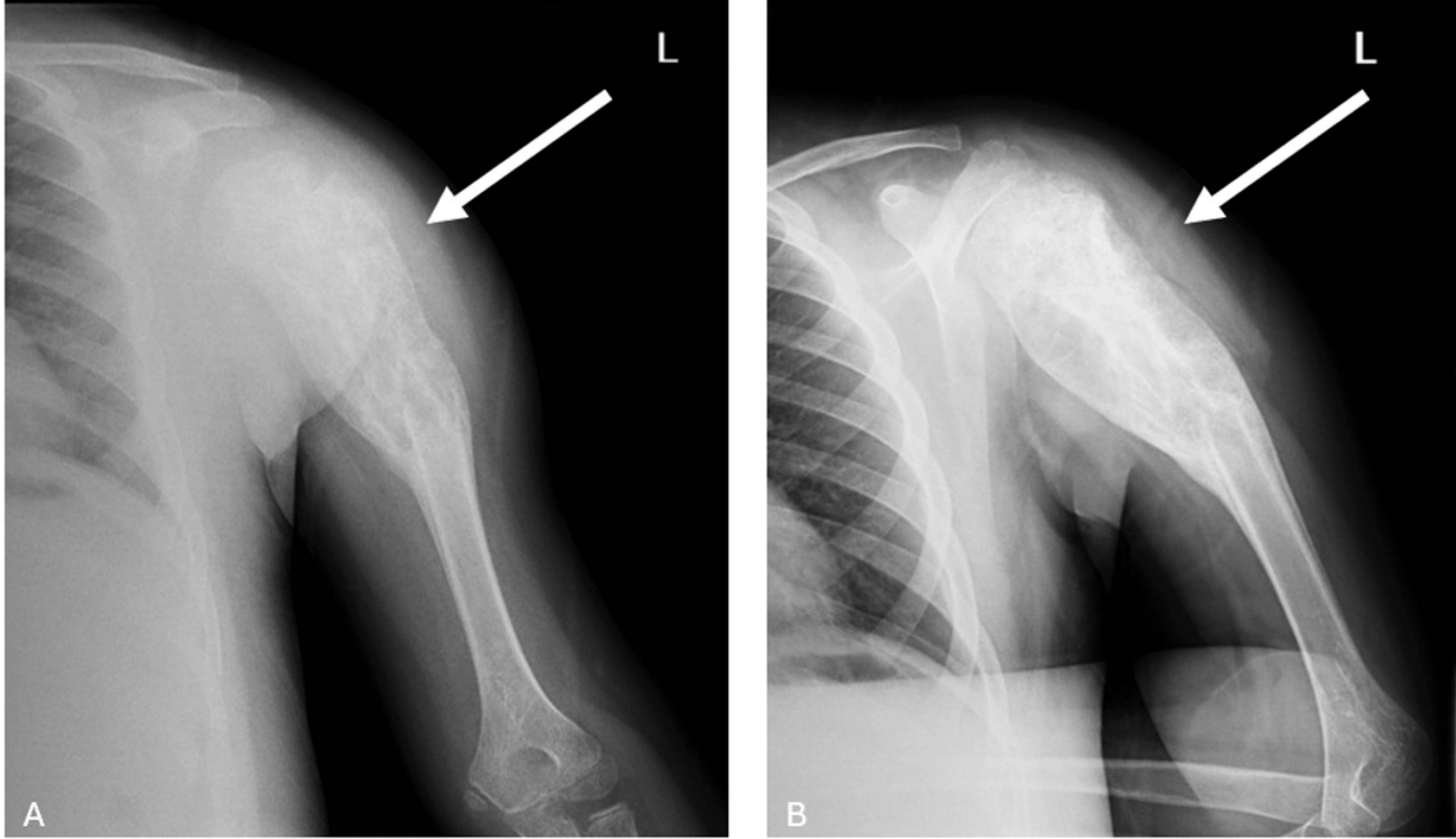
Preoperative X-ray images of the patient at admission (A) Anteroposterior (AP) view of the shoulder and (B) lateral view of the humerus are shown. White arrows indicate the lesion at the proximal humerus. The radiographs revealed an expansile, lytic lesion with cortical thinning and endosteal scalloping, consistent with a locally aggressive bone lesion

An eight-year-old male patient, 137 cm tall and weighing 52 kg, presented to our clinic with complaints of swelling and pain in the left shoulder. The patient had no axillary or pubic hair development and was classified as Tanner stage 1. Axillary region examination revealed a metastatic lymphadenopathy. Informed consent was obtained before any procedures. The patient had no known additional health issues, was not on any regular medication, and had no history of prior surgeries. On general examination, the patient was well-appearing and afebrile, with stable vital signs and no systemic signs of illness. A palpable swelling was noted on the lateral side of the left arm, measuring approximately 3x4 cm, which was firm and mildly tender to palpation. There were no signs of inflammation, such as redness, warmth, or swelling, and no visible ulceration was present over the skin. Joint and range of motion assessments were nearly normal, and the axillary nerve examination was unremarkable. Radiological imaging was subsequently requested (Figure 1).

**Figure 2:**
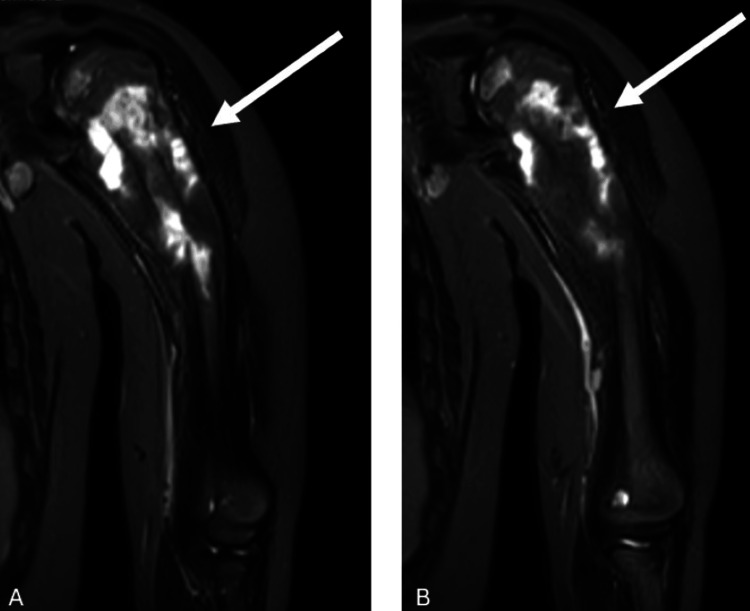
Preoperative sagittal MRI images of the patient at admission (A-B) The images show the lesion at the proximal humerus, indicated by white arrows. In (B), the distal involvement was evaluated by the tumor board and was not considered skip metastasis MRI: magnetic resonance imaging

**Figure 3:**
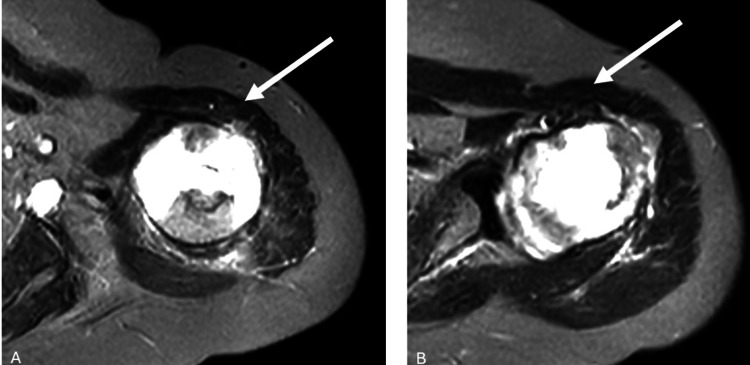
Axial MRI section images of the patient (A-B) The images demonstrate an intramedullary osteosarcoma located in the proximal humerus. The white arrows indicate the region affected by osteosarcoma MRI: magnetic resonance imaging

**Figure 4:**
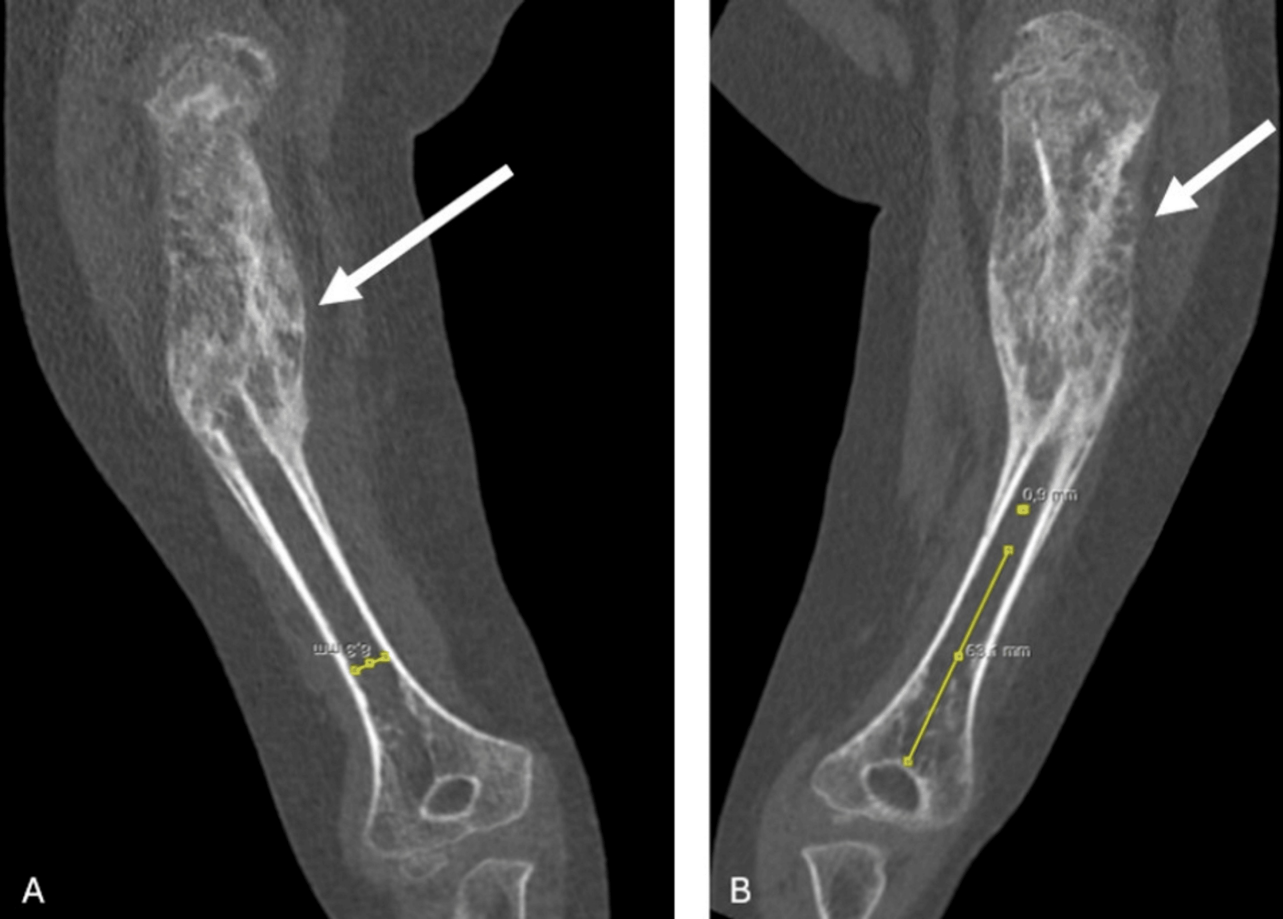
Coronal MRI section images of the patient (A) The canal diameter of the humerus was measured at 8.3 mm. (B) The humeral resection margin was assumed to measure 63.5 mm. The white arrows indicate the region affected by osteosarcoma MRI: magnetic resonance imaging

X-rays revealed a mass in the proximal humerus, prompting a contrast-enhanced MRI. MRI findings described a mass measuring 95 × 46 × 44 mm, consistent with osteosarcoma. The measured canal diameter was 8.3 mm, and the predicted humeral resection margin was estimated to be 63.5 mm from the level of the capitellum (Figures 2-4).

PET-CT revealed no metabolically active lesion except the axillary lymph node with SUV 8.7. A tru-cut biopsy confirmed a high-grade intramedullary osteosarcoma, classified as Stage III according to both the Enneking and Musculoskeletal Tumor Society (MSTS) staging systems. The patient’s immunohistochemical staining showed the following findings: CD99: positive, NKX2-2: positive, and SATB2: positive in scattered cells. The patient underwent four cycles of neoadjuvant chemotherapy with ifosfamide, epirubicin, and cisplatin and was scheduled for two additional cycles of adjuvant chemotherapy postoperatively.

**Figure 5:**
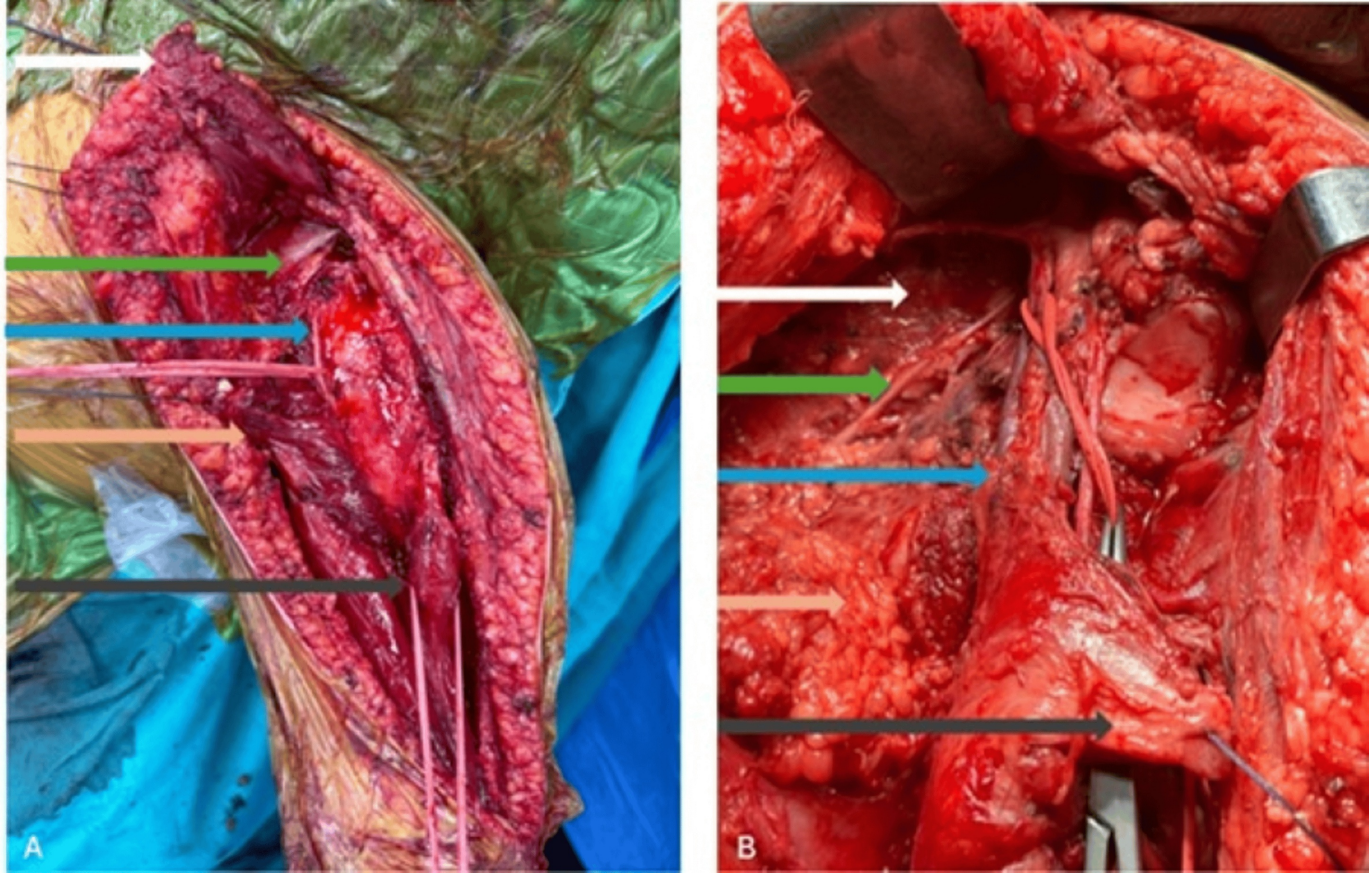
Intraoperative clinical images - 1 (A) Tumor bed after wide resection: white arrow, pectoralis major; green, pectoralis minor; blue, musculocutaneous nerve; brown, coracobrachialis; gray, long head of biceps brachii. (B) Post–axillary lymph node dissection: white, thoracic wall; green, long thoracic nerve; blue, axillary vessels and nerves; brown, dissected lymph node; gray, coracobrachialis

**Figure 6:**
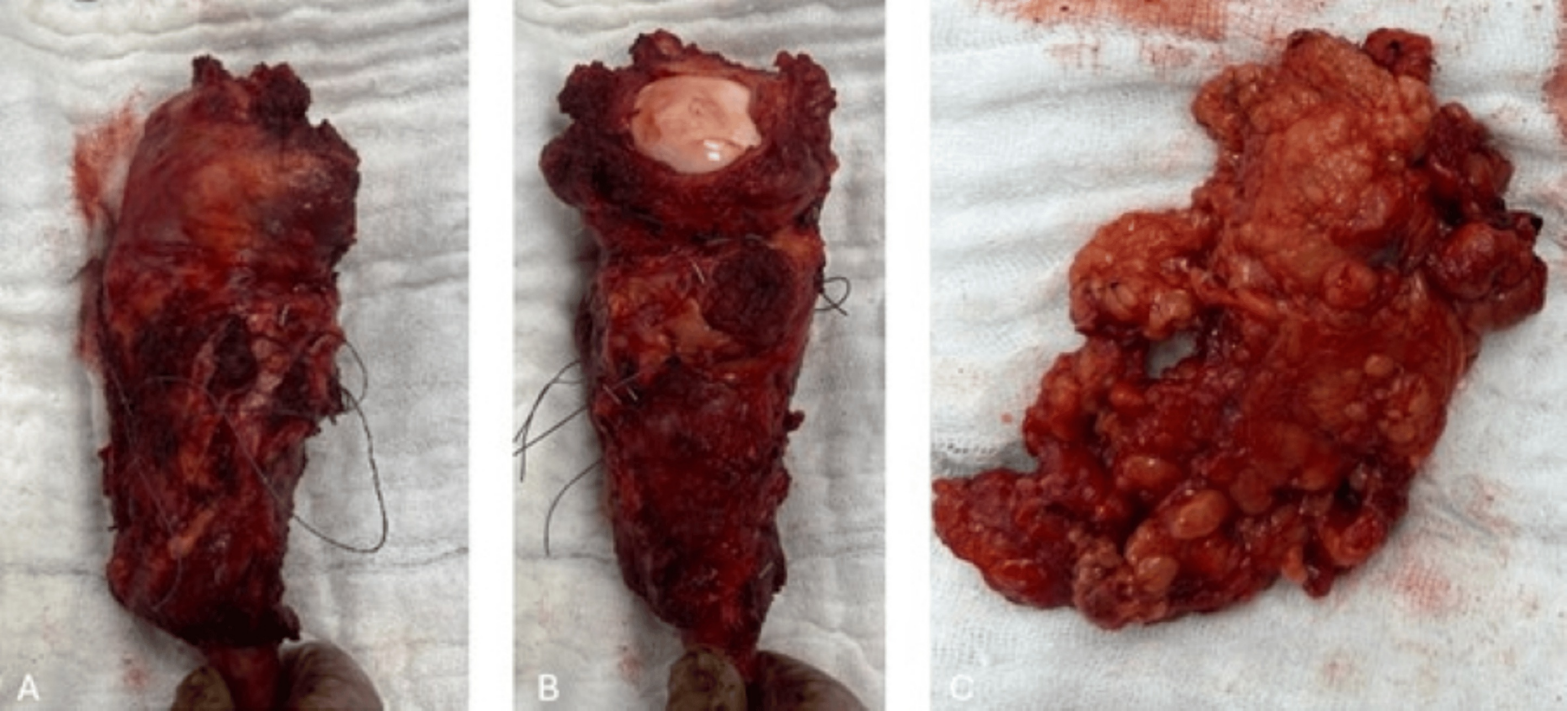
Intraoperative image of the resected mass (A) Anterior view of the lesion. (B) Posterior view of the lesion. (C) Excised axillary lymph node after resection

**Figure 7:**
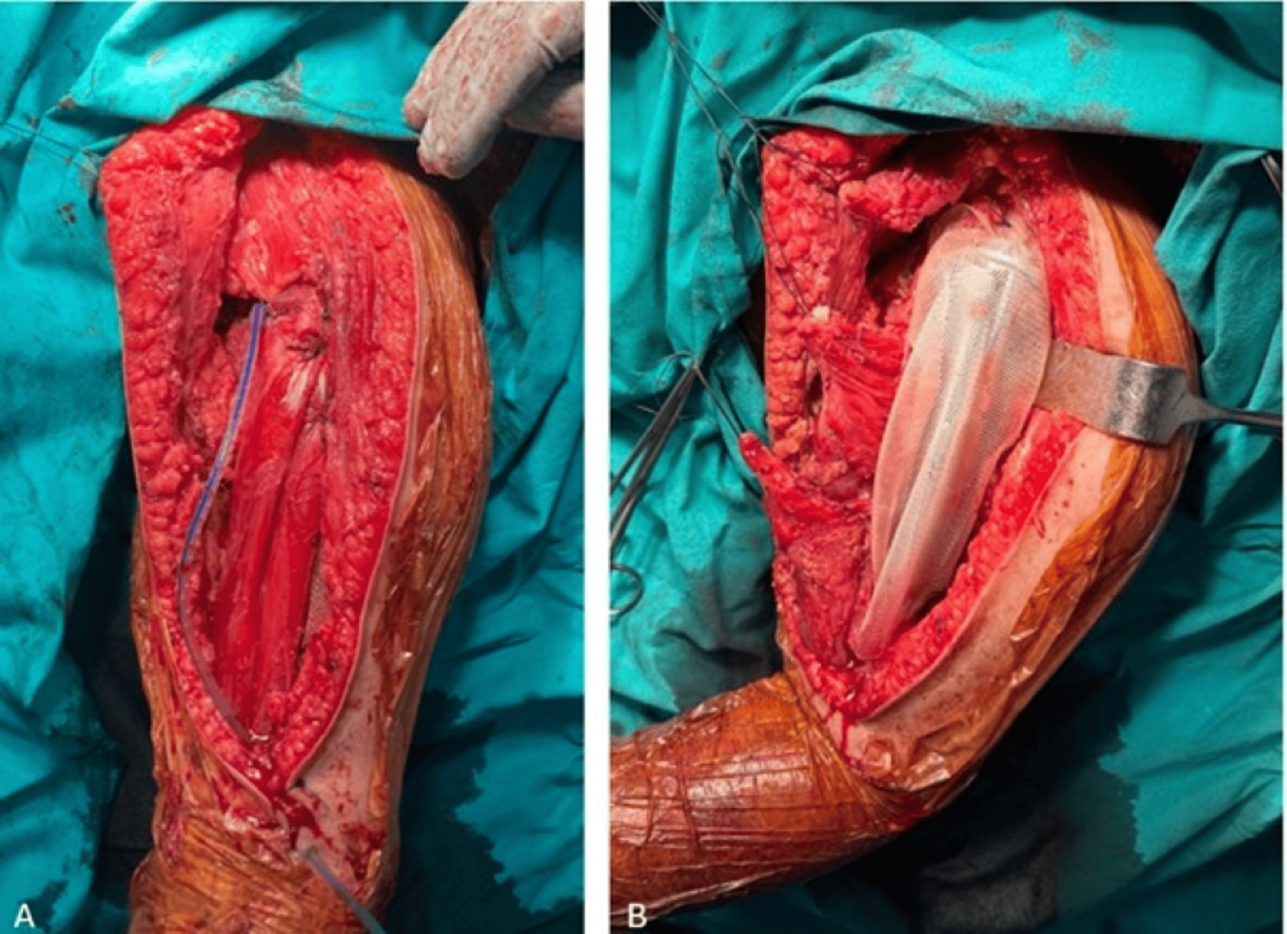
Intraoperative clinical images - 2 (A) Image of the surgical site after drain placement, showing the area prepared for prosthesis implantation. (B) Image of the surgical site before prosthesis placement, reinforced with Prolene mesh

The case was reviewed at a tumor board meeting, and the treatment plan included an open biopsy, followed by chemotherapy, wide resection, and reconstruction with a tumor resection prosthesis. Axillary lymph node dissection was performed during surgery (Figures 5-7), and frozen section analysis was conducted.

As the frozen results were negative, reconstruction was carried out using a tumor resection prosthesis with the assistance of Prolene mesh after the wide resection. The duration of the surgery was 12 hours in total.

**Figure 8:**
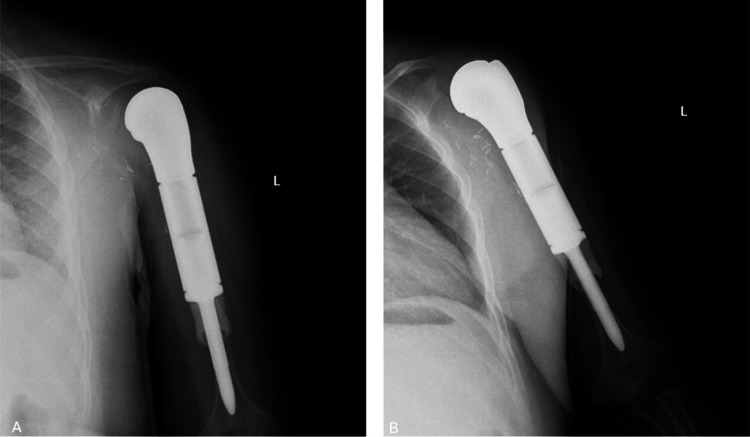
Postoperative X-ray images of the patient at check-up at 1.5 year postoperatively (A) Humerus anteroposterior (AP) view. (B) Humerus lateral view

The pathological diagnosis was reported as high-grade intramedullary osteosarcoma. Immunohistochemistry showed CD99-positive, NKX2-2-positive, and SATB2 was positive in scattered cells. Surgical margins were free of tumor, and regional lymph node biopsy was negative for metastasis. The patient received four cycles of chemotherapy before surgery and two cycles after surgery, consisting of ifosfamide, epirubicin, and cisplatin. Adjuvant chemotherapy was initiated without delay, beginning approximately 14 days after surgery. During routine postoperative follow-ups, no wound complications were observed. At 1.5 years postoperatively, the prosthesis remained intact, and no metastases were identified (Figure 8).

**Figure 9:**
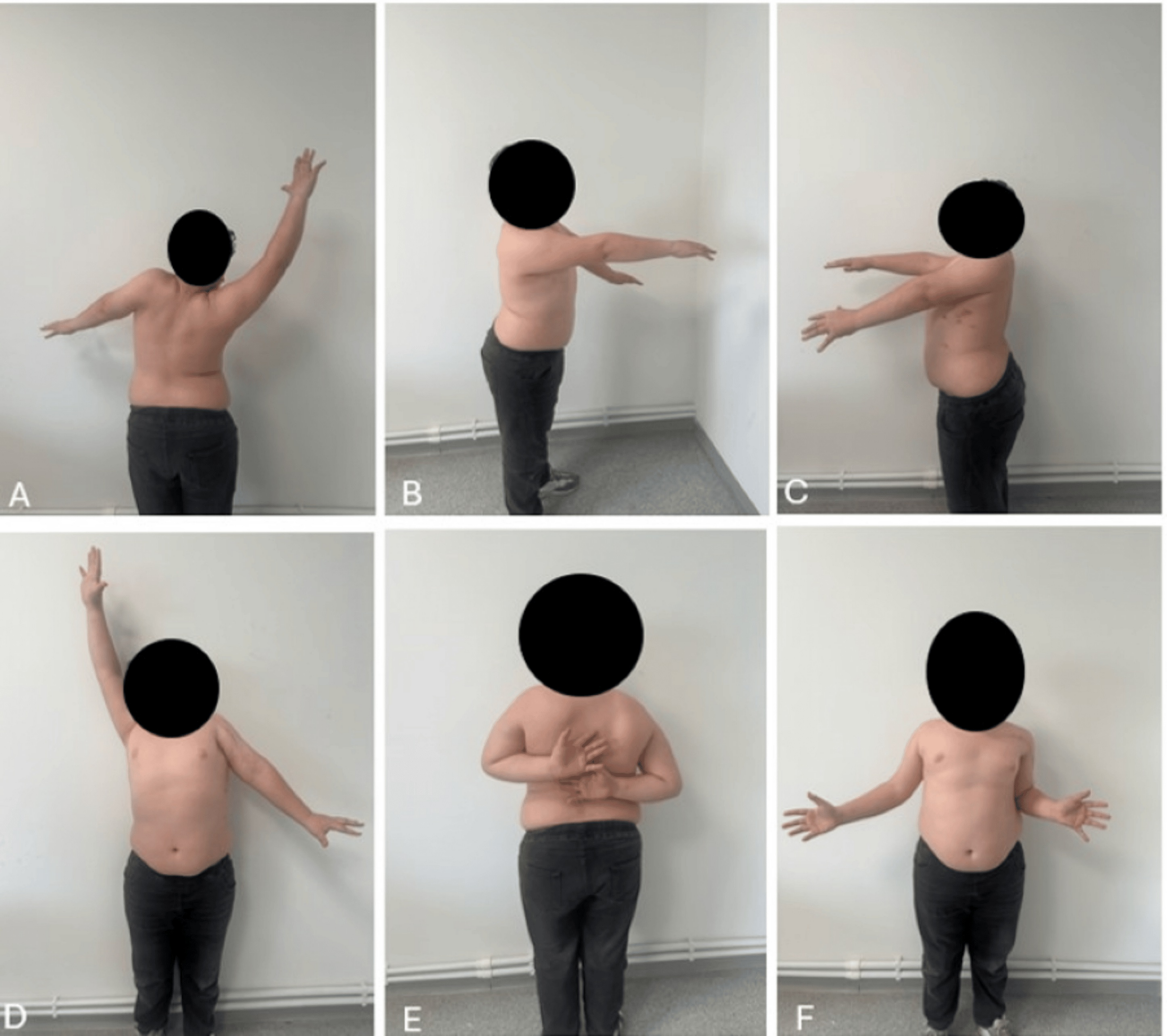
Postoperative, 18-month follow-up joint range of motion of the patient (A, B, and C) Forward elevation of the shoulder is observed to be 80 degrees. (D) Lateral elevation is observed to be 45 degrees. (E) Internal rotation is observed to be near full. (F) External rotation is also observed to be near full

At 1.5 years postoperatively, the shoulder on the operated side showed 80 degrees of forward flexion, 50 degrees of lateral elevation, nearly full external rotation, and restricted internal rotation (Figure 9).

## Discussion

Osteosarcoma of the proximal humerus presents unique diagnostic and therapeutic challenges due to its anatomical location and functional significance in upper limb mobility. Although osteosarcoma predominantly affects the metaphyseal regions of long bones, it occurs in the proximal humerus in approximately 10-15% of cases. This site is particularly challenging for surgical resection due to its proximity to vital neurovascular structures and the difficulty of achieving limb preservation without compromising function [7]. Our patient, an eight-year-old male, presented with swelling and pain in the left shoulder. Imaging studies confirmed a proximal humeral mass with features suggestive of osteosarcoma. PET-CT revealed no metabolically active lesion except the axillary lymph node with SUV 8.7. Although axillary lymph node metastasis is uncommon in osteosarcoma, its presence may indicate an aggressive disease course and requires careful consideration in treatment planning.

Current treatment protocols for osteosarcoma rely on a multimodal approach combining neoadjuvant chemotherapy, surgical resection, and adjuvant chemotherapy. It is acknowledged that a methotrexate-based regimen could also have been an appropriate option. The standard chemotherapy regimen includes ifosfamide, epirubicin, and cisplatin, which has been shown to improve overall survival by targeting micrometastatic disease [8]. Our patient was treated with a chemotherapy regimen consisting of ifosfamide, epirubicin, and cisplatin, administered over four cycles preoperatively and two cycles postoperatively, which likely contributed to tumor size reduction and facilitated wide resection with negative margins (Table 1).

**Table 1:** Reconstruction options based on clinical scenarios APC: allograft-prosthetic composite

Nerve preservation status	Age group	Reconstruction options
Deltoid and axillary nerve preserved	Juvenile (intraarticular)	Vascularized fibula with head (anterior tibial artery)
Deltoid and axillary nerve preserved	Juvenile (extraarticular)	Fusion with vascularized fibula (peroneal artery), scapular pillar graft, clavicula pro humero, or vascular fibula with head (anterior tibial artery)
Deltoid and axillary nerve preserved	Adolescent (intraarticular)	Rotator cuff repairable: vascular fibula with head (anterior tibial artery), reverse polarity endoprosthesis, APC, or endoprosthesis + mesh
Deltoid and axillary nerve preserved	Adolescent (extraarticular)	Vascular fibula with head (anterior tibial artery), reverse polarity endoprosthesis, or reverse APC
Deltoid and axillary nerve not preserved	Any age	Fusion with isolated vascularized fibular free flap (peroneal artery) or scapular pillar graft ± allograft
Deltoid and axillary nerve not preserved	<6 years old	Vascular fibula with head (anterior tibial artery)
Deltoid and axillary nerve not preserved	Adolescent (extraarticular)	Reverse polarity endoprosthesis, fusion, or vascular fibula with head (and medial buttress)

Surgical treatment of osteosarcoma involves various complications influenced by the type of reconstruction and extent of surgery. The key challenges include local recurrence, systemic metastasis (especially to the lungs), and infections that may occur early or late in the postoperative period. In growing children, limb shortening following resection can lead to functional and aesthetic concerns [9]. Prosthetic reconstructions also carry risks such as aseptic loosening, stress fractures, wear of polyethylene components, and pathological fractures, often requiring further interventions [10].

Limb-salvage surgery has become the preferred surgical strategy for osteosarcoma of the proximal humerus, replacing historical amputation approaches [11]. However, resection in this region presents technical challenges due to its proximity to the axillary neurovascular bundle [12-13]. In our case, wide resection was performed along with axillary lymph node dissection, which is rarely reported in the literature, given the uncommon occurrence of lymphatic spread in osteosarcoma. Reconstruction was performed using a tumor resection prosthesis, and a polypropylene mesh was applied for additional stabilization. The mesh served to reinforce the soft tissue envelope and improve prosthetic integration, particularly after extensive bone and soft tissue loss. At 18 months postoperatively, the patient demonstrated satisfactory functional outcomes without evidence of local recurrence or distant metastasis.

The long-term prognosis of osteosarcoma depends on several factors, including tumor size, histological response to chemotherapy, surgical margins, and metastatic status at presentation [14]. The presence of axillary lymph node involvement in our patient raises concerns about the potential for disease progression, although its impact on prognosis remains uncertain due to limited data on lymphatic spread in osteosarcoma [15]. Close follow-up with serial imaging is essential to monitor for recurrence or metastasis. 

A systematic review by Groundland et al. emphasized the importance of selecting appropriate reconstruction strategies in pediatric limb-sparing surgery. The study highlighted that proper surgical planning improves functional outcomes, reduces amputation rates, and enhances the quality of life. Comparative data on metallic endoprostheses, allografts, and composite reconstructions revealed variations in MSTS scores and range of motion, underscoring the need to individualize treatment plans. The authors concluded that standardized data and advancements in prosthetic design are essential to further improve outcomes in pediatric patients with bone tumors [13].

Despite advances in treatment, optimizing functional outcomes and long-term survival in patients with proximal humeral osteosarcoma remains challenging. Reconstruction strategies for the proximal humerus vary, each with distinct advantages and limitations. Biological reconstructions, such as autografts and allografts, are preferred for younger patients due to their ability to integrate with native bone and support functional recovery. However, these methods involve longer surgeries and are associated with risks like donor-site morbidity and mechanical complications. Prosthetic options, including anatomical and reverse shoulder prostheses, provide shorter surgical times and quicker recovery, making them suitable for older patients, though their durability may be limited over time. Composite reconstructions, combining biological and prosthetic techniques, offer favorable outcomes but are technically demanding and require extended surgical time. The integration of novel therapies, such as targeted treatments and immunotherapy, holds promise for improving disease control and reducing recurrence rates. Additionally, future research on the prognostic impact of axillary lymph node involvement in osteosarcoma is crucial for enhancing treatment strategies and refining risk stratification.

## Conclusions

This report underscores the importance of a multidisciplinary approach in managing proximal humeral osteosarcoma, highlighting the role of neoadjuvant chemotherapy, careful surgical planning, and individualized reconstruction. While short-term outcomes were favorable, ongoing surveillance is essential for long-term disease control and function. Adult-style reconstruction may work in young adults but is often less suitable for smaller children. For pediatric patients, minimizing revisions and reducing reliance on endoprostheses is ideal. Combining biological reconstruction with tendon or nerve transfers, often in collaboration with plastic surgeons, demands time and expertise but can yield meaningful long-term benefits.
